# Government policy interventions to reduce human antimicrobial use: protocol for a systematic review and meta-analysis

**DOI:** 10.1186/s13643-017-0640-2

**Published:** 2017-12-13

**Authors:** Susan Rogers Van Katwyk, Jeremy M. Grimshaw, Marc Mendelson, Monica Taljaard, Steven J. Hoffman

**Affiliations:** 1 School of Epidemiology and Public Health, University of Ottawa, Ottawa, ON Canada; 2 Global Strategy Lab, Dahdaleh Institute for Global Health Research, Faculty of Health and Osgoode Hall Law School, York University, Ottawa, ON Canada; 3 Clinical Epidemiology Program, Ottawa Hospital Research Institute, Ottawa, ON Canada; 4 Department of Medicine, University of Ottawa, Ottawa, ON Canada; 5 Division of Infectious Diseases and HIV Medicine, Groote Schuur Hospital, University of Cape Town, Cape Town, South Africa; 6 Department of Health Research Methods, Evidence, and Impact, McMaster Health Forum, McMaster University, Hamilton, ON Canada; 7 Department of Global Health and Population, Harvard T.H. Chan School of Public Health, Harvard University, Boston, MA USA

**Keywords:** AMR, Antimicrobial resistance, Policy, Government, Systematic review, Protocol

## Abstract

**Background:**

Antimicrobial resistance (AMR) is a recognized threat to global public health. Increasing AMR and a dry pipeline of novel antimicrobial drugs have put AMR in the international spotlight. One strategy to combat AMR is to reduce antimicrobial drug consumption. Governments around the world have been experimenting with different policy interventions, such as regulating where antimicrobials can be sold, restricting the use of last-resort antimicrobials, funding AMR stewardship programs, and launching public awareness campaigns. To inform future action, governments should have access to synthesized data on the effectiveness of large-scale AMR interventions. This planned systematic review will (1) identify and describe previously evaluated government policy interventions to reduce human antimicrobial use and (2) estimate the effectiveness of these different strategies.

**Methods:**

An electronic search strategy has been developed in consultation with two research librarians. Seven databases (MEDLINE, CINAHL, EMBASE, CENTRAL, PAIS Index, Web of Science, and PubMed excluding MEDLINE) will be searched, and additional studies will be identified using several gray literature search strategies. To be included, a study must (1) clearly describe the government policy and (2) use a rigorous design to quantitatively measure the impact of the policy on human antibiotic use. The intervention of interest is any policy intervention enacted by a government or government agency in any country to change human antimicrobial use. Two independent reviewers will screen for eligibility using criteria defined a priori.

Data will be extracted with Covidence software using a customized extraction form. If sufficient data exists, a meta-analysis by intervention type will be conducted as part of the effectiveness review. However, if there are too few studies or if the interventions are too heterogeneous, data will be tabulated and a narrative synthesis strategy will be used.

**Discussion:**

This evidence synthesis is intended for use by policymakers, public health practitioners, and researchers to inform future government policies aiming to address antimicrobial resistance. This review will also identify gaps in the evidence about the effectiveness of different policy interventions to inform future research priorities.

**Systematic review registration:**

PROSPERO CRD42017067514.

**Electronic supplementary material:**

The online version of this article (10.1186/s13643-017-0640-2) contains supplementary material, which is available to authorized users.

## Background

Antimicrobial resistance (AMR) is widely recognized as a serious threat to global public health. One estimate suggests that resistance is currently responsible for 700,000 deaths per year, a death toll that is projected to reach 10 million a year by 2050 [1]. The potential for AMR has been recognized since the earliest days of antibiotics [2]; yet, the misuse and overuse of antimicrobials have persisted over decades, contributing to the development of resistance.

Overuse of antibiotics exerts a selection pressure on microbes and accelerates the evolution of resistant strains of disease-causing organisms [3]. In the past, the threat of AMR was addressed by developing novel antimicrobial agents; however, there have not been any such novel agents developed in over 20 years, and few new potential agents are in development [4–6]. As a result, there is increasing awareness around the need to minimize the development of AMR through better conserving the effectiveness of existing antimicrobials.

AMR is a complex and multi-faceted problem, and intervening to minimize resistance is difficult. A key part of the global strategy on AMR is promoting the appropriate use of antimicrobials; inappropriate use and overuse of antimicrobials are major drivers of resistance [3]. Evidence suggests that reducing antibiotic use is associated with lower rates of resistance [3]. Two systematic reviews have linked community antimicrobial use to resistance; one found that individuals prescribed an antibiotic in primary care developed resistance to that antibiotic with detectable effects for up to a year [7], while the other found higher resistance rates among pathogens circulating in areas with higher antibiotic use [8]. Mathematical modeling suggests that reductions in antibiotic use would result in lower resistance rates because genetically less “fit” resistant strains would be outcompeted by susceptible strains [9]. Recent analyses of a Scottish intervention found substantial decreases in methicillin-resistant *Staphylococcus aureus* (MRSA) and *Clostridium difficile* infections associated with decreases in prescribing of key antibiotics [10, 11]. Reducing antimicrobial use among humans is important given the relationship between hospital and community infections; the same Scottish study found that prevalence of MRSA in the community was predicted by hospital MRSA rates. Stewardship activities within the community were associated with a 32% reduction in community MRSA, while hospital-based interventions were responsible for an additional 37% reduction in community MRSA [11].

Reducing antimicrobial use is, therefore, one of the most common strategies that has been pursued to reduce AMR, and antimicrobial use is one of the most commonly measured intervention evaluation indicators. Many hospitals, for example, have implemented AMR stewardship programs to reduce physician prescribing [12]. Governments—which have various policy levers at their disposal—have also had success at reducing antimicrobial use. Mexico [13], Brazil [14], and Chile [15], for example, all saw reductions in antibiotic use after implementing policies to prevent over-the-counter sales of antibiotics. Less direct interventions have also reduced antibiotic use; in Canada, a policy to provide free universal influenza immunization was associated with a 64% reduction in inappropriate antibiotic prescriptions for influenza [16].

Using government policy to promote public health is generally well-worth considering when challenges require widespread and uniform compliance with a defined set of minimum standards [17]; in that vein, research to date suggests that government-level intervention may play a key role in reducing AMR [18]. Currently, however, global antimicrobial use is increasing; between 2000 and 2010, there was a 36% global increase in antibiotic use, 75% of which came from five of the world’s fastest growing economies: Brazil, Russia, India, China, and South Africa [19].

While the importance of government-level intervention is clear, to our knowledge, there has not yet been a systematic review evaluating the extent to which different government policy interventions successfully reduce antimicrobial use. This gap is worth filling urgently considering that many governments around the world are actively considering numerous types of policy interventions that could reduce antimicrobial use, utilizing policy levers such as legislation, taxation, economic incentives, funding support, public awareness campaigns, and regulation of professionals and businesses whose work might affect AMR [17]. The magnitude of AMR’s combined threat to human health and economic development [1] is already incentivizing governments to take a greater role in addressing AMR. Given the World Health Organization’s recent call for the development of National Action Plans on AMR [20], a focus on the potential impact of government policy interventions on antimicrobial use is extremely timely. In light of these motivations and challenges, this systematic review intends to support evidence-informed action on AMR at the government level, by identifying, describing, and assessing the impact of government policy interventions on human antimicrobial use. More specifically, this systematic review aims to produce the following: (1) a *descriptive review* that identifies and describes the government policy interventions that have been implemented and evaluated with the objective of changing antimicrobial use in humans and (2) an *effectiveness review* that assesses the impact of these government policy interventions on reducing human antimicrobial use.

## Methods

### Protocol and registration

This systematic review protocol has been reported in accordance with the Preferred Reporting Items for Systematic Review and Meta-Analysis Protocols (PRISMA-P) guidelines [21] (see details in Additional file 1). Our review will be carried out in accordance with this protocol, and details of any changes to this protocol will be reported in the final review manuscript. This study has been registered in PROSPERO, the International Prospective Register of Systematic Reviews (Registration number CRD42017067514).

### Criteria for including studies in this review

#### Types of study designs

For the descriptive review, we will include any study that quantitatively describes the impact of a government policy intervention to reduce inappropriate antimicrobial use. Inclusion of all quantitative study designs allows us to identify a broad range of policy interventions and facilitates our goal of identifying and describing the government policy interventions to address antimicrobial consumption that have been implemented and evaluated to date (Table 1).

**Table 1:** Summary of inclusion/exclusion criteria for phase 1 and phase 2 of screening

	Criteria	Include if	Exclude if
Phase 1/title and abstract screening	1) The study assesses a primary outcome measure of interest.	Study reports on *human antimicrobial use*, including consumption, prescribing, dosing, and sales of an antibiotic, antiviral, antiparasitic, or antifungal drug.	Study does not report on antimicrobial use or only reports on antimicrobial use in animals or agriculture.
	2) The study quantitatively evaluates the effect of an intervention.	Study is a *quantitative impact evaluation*.	Study is a qualitative study, editorial, commentary, review, or synthesis or does not evaluate the impact of an intervention.
	3) A policy intervention is evaluated.	A *policy intervention* using education, persuasion, incentivization, coercion, training, restriction, changing the physical or social context, modeling appropriate behavior, or reducing barriers to action is being evaluated.	Intervention was not a change to a policy (e.g., clinical study).
Phase 2/full-text screening	1) The study assesses a primary outcome measure of interest.	Study reports on *human antimicrobial use*, including consumption, prescribing, dosing, and sales of an antibiotic, antiviral, antiparasitic, or antifungal drug.	Study does not report on antimicrobial use or only reports on antimicrobial use in animals or agriculture.
	2) The study quantitatively evaluates the effect of an intervention.	Study is a *quantitative impact evaluation*.	Study is a qualitative study, editorial, commentary, review, or synthesis or does not evaluate the impact of an intervention.
	3) The study reports on a policy intervention enacted at the government level.	The policy intervention was *enacted by a government or government agency* at the federal, state, provincial, or municipal level.	Intervention was not enacted by a government (e.g., intervention was enacted in a single hospital or network of hospitals).
	4) The intervention is clearly described	The study provides a description that includes the intervention’s aim, enacting government authority, timing, and form.	The study does not describe the aim, governing body, timing of the intervention, or form of the intervention.
	Effectiveness review only: 5) The study design is sufficiently rigorous to meet the minimum methodological requirements of the Cochrane Collaboration’s EPOC group	Effectiveness review only: Study design is experimental or quasi-experimental, such as randomized controlled trial, interrupted time series, and controlled before/after study, or it can be re-analyzed to meet this standard.	Effectiveness review only: Study does not have a control group or use pre-intervention data and cannot be re-analyzed.

For the effectiveness review, our goal is to assess the impact of government policy interventions on the quantity of antimicrobials consumed by humans. We will include experimental designs like randomized controlled trials (including stepped wedge designs) and quasi-experimental designs like interrupted time-series analyses and controlled before-and-after studies. Quasi-experimental designs will need to meet the minimum methodological requirements of the Cochrane Collaboration’s Effective Practice and Organization of Care (EPOC) group. This means that all controlled studies, including controlled before-and-after studies, randomized controlled trials, and non-randomized controlled trials, must have at least two intervention and two control sites and interrupted time-series studies must have at least three measurements pre- and post-intervention to be included. Uncontrolled before/after studies with at least three measurements pre- and post-intervention may also be included if it is possible to re-analyze the data using an interrupted time-series analysis. We anticipate most included studies will evaluate impact using routinely collected data from before and after the intervention’s implementation; a recent review of government policy interventions addressing dietary sodium consumption found this to be the most popular study design [22]. Qualitative studies, editorials, commentaries, reviews, and other syntheses will be excluded; however, the reference lists of relevant reviews and syntheses will be hand-searched to identify other potentially relevant studies.

#### Types of participants

The impact of a government policy may be evaluated at any level, from the population level to the single hospital or clinic level; for example, a study investigating the impact of a national guideline might evaluate the impact on antibiotic sales at a national level, but it could also evaluate the impact on prescribing rates in a single hospital. However, in their statistical analysis, studies may include data from anyone involved in the sale, purchase, prescription, or consumption of antimicrobial drugs such as patients, health professionals, hospitals, pharmacies, and other healthcare facilities. Studies that evaluate interventions in any country or simultaneously in multiple countries (e.g., European Union) are eligible for inclusion.

#### Types of interventions

Any policy intervention enacted by a government to reduce human antimicrobial use will be included. Interventions may be population-based, or targeted to specific groups such as health professionals. To be included, a study must clearly describe the government policy by providing a description that includes the intervention’s aim, enacting government authority, timing, and form.

Globally, many levels of government have jurisdiction over health issues. As such, we will follow an inclusive approach to defining “government.” A study is eligible for inclusion if the evaluated policy intervention is enacted by any level of government, including national, state, provincial, regional, and municipal governments, or by a government-controlled agency, ministry, or department. Studies evaluating interventions developed by individual hospitals, pharmacies, and other healthcare facilities will not be included, even if these healthcare facilities are owned, operated, governed, or otherwise controlled by governments. As such, a study evaluating the impact of an audit and feedback program developed by a single hospital or even a network of healthcare facilities would not be eligible.

Policy interventions will be defined in accordance with the Behavior Change Wheel framework; as such, policy interventions are defined as those that create change in antimicrobial use through education, persuasion, incentivization, coercion, training, restriction, changing the physical or social context, modeling appropriate behavior, or reducing barriers to action [23]. Examples of such policy interventions at the government level would include the following: regulating where antimicrobials can be sold, restricting the use of last-resort antibiotics, or launching public awareness campaigns. Comparators or control groups may include the absence of the government policy or comparison with another policy. Eligible interventions can target all antimicrobials (e.g., all antibiotics), specific antimicrobials, or particular groups of antimicrobials (e.g., 4C antibiotics).

#### Types of outcomes

Many countries do not collect sufficient regional or national surveillance data on AMR to allow analysis of the impact of policy-level interventions on resistance, and surveillance definitions of resistance vary between countries [24]. As such, we chose to focus on antimicrobial use, which is an intermediary between policy interventions and AMR. Better data exists for this outcome, as prescribing and sales of antibiotics are often captured as routine health administrative data. Relevant outcome measures of antimicrobial use include self-reported antimicrobial consumption, measured antimicrobial consumption, antimicrobial prescribing, antimicrobial dosing, and/or antimicrobial sales. Secondary outcomes will include any other reported beneficial or adverse outcomes related to the intervention. These secondary outcomes might include, for example, changes in rates of AMR, or increases and decreases in the rates of hospital re-admission or return clinic visits, resulting from a government policy intervention to reduce human antimicrobial consumption.

### Information sources

Seven electronic databases will be searched: MEDLINE, CINAHL, EMBASE, PAIS Index, Cochrane Central Register of Controlled Trials (CENTRAL), and Web of Science from inception to March 2017. PubMed will also be searched for relevant articles that are not indexed in MEDLINE, using the search filter developed by the Canadian Agency for Drugs and Technologies in Health [25]. The MEDLINE search strategy is available in Table 2; the search strategies for all databases are in Additional file 2. The search strategies for the four biomedical databases (MEDLINE, EMBASE, CENTRAL, and PubMed) include search terms for 172 antibiotics names; this list of antibiotics was used in the recent Cochrane review of interventions to improve antibiotic prescribing in hospitals [12]. The search strategies for the social science and allied health databases (CINAHL, PAIS Index, Web of Science) included the generic terms “antibiotic” and “antimicrobial” instead of specific antibiotic names. No date or language limits have been applied; we will endeavor to translate studies that are not in languages spoken by members of the study team (i.e., English and French). To identify gray literature, we will use keywords to conduct targeted web searching to identify government and civil society reports and hand-search reference lists of included studies to identify non-indexed articles. We will also use the ProQuest Dissertations and Theses database to identify dissertations on this topic. After full-text screening, we will also contact six subject-matter experts—one from each WHO region—to identify any additional studies from their region that meet our inclusion criteria. This search strategy was designed in consultation with a health science research librarian and a government information research librarian at the University of Ottawa. The search strategy has been peer-reviewed using PRESS [26].

**Table 2:** MEDLINE search strategy

Theme	Search terms
Antibiotic use	1. Anti-Bacterial Agents/
	2. Anti-infective Agents/
	3. 1 or 2
	4. ((antibiotic? or alamethicin? or amdinocillin? or amdinocillin pivoxil? or amikacin? or amoxicillin? or amoxicillin-potassium clavulanate combination? or amphotericin? or ampicillin? or anisomycin? or antimycin? or aurodox? or azithromycin? or azlocillin? or aztreonam? or bacitracin? or bacteriocin? or bambermycin? or bongkrekic acid? or brefeldin? or butirosin sulfate? or calcimycin? or candicidin? or capreomycin? or carbenicillin? or carfecillin? or cefaclor? or cefadroxil? or cefamandole? or cefatrizine? or cefazolin? or cefixime? or cefmenoxime? or cefmetazole? or cefonicid? or cefoperazone? or cefotaxime? or cefotetan? or cefotiam? or cefoxitin? or cefsulodin? or ceftazidime? or ceftizoxime? or ceftriaxone? or cefuroxime? or cephacetrile? or cephalexin? or cephaloglycin? or cephaloridine? or cephalosporin? or cephalothin? or cephamycin? or cephapirin? or cephradine? or chloramphenicol? or chlortetracycline? or citrinin? or clarithromycin? or clavulanic acid? or clavulanic acid? or clindamycin? or cloxacillin? or colistin? or cyclacillin? or dactinomycin? or daptomycin? or demeclocycline? or dibekacin? or dicloxacillin? or dihydrostreptomycin sulfate? or diketopiperazine? or distamycin? or doxycycline? or echinomycin? or edeine? or enviomycin? or erythromycin? or erythromycin estolate? or erythromycin ethylsuccinate? or filipin? or floxacillin? or fluoroquinolone? or fosfomycin? or framycetin? or fusidic acid? or gentamicin? or gramicidin? or hygromycin? or imipenem? or josamycin? or kanamycin? or kitasamycin? or lactam? or lasalocid? or leucomycin? or lincomycin? or lincosamide? or lucensomycin? or lymecycline? or mepartricin? or methacycline? or methicillin? or mezlocillin? or mikamycin? or minocycline? or miocamycin? or moxalactam? or mupirocin? or mycobacillin? or nafcillin? or natamycin? or nebramycin? or neomycin? or netilmicin? or netropsin? or nigericin? or nisin? or norfloxacin? or novobiocin? or nystatin? or ofloxacin? or oleandomycin? or oligomycin? or oxacillin? or oxytetracycline? or paromomycin? or penicillanic acid? or penicillic acid? or penicillin?? or piperacillin? or pivampicillin? or polymyxin b? or polymyxin? or pristinamycin? or prodigiosin? or ribostamycin? or rifabutin? or rifamycin? or ristocetin? or rolitetracycline? or roxarsone? or roxithromycin? or rutamycin? or sirolimu? or sisomicin? or spectinomycin? or spiramycin? or streptogramin?? or streptomycin? or streptovaricin? or sulbactam? or sulbenicillin? or sulfamerazine? or sulfamethoxypyridazine? or talampicillin? or teicoplanin? or tetracycline? or thiamphenicol? or thienamycin? or thiostrepton? or ticarcillin? or tobramycin? or troleandomycin? or tunicamycin? or tylosin? or tyrocidine? or tyrothricin? or valinomycin? or vancomycin? or vernamycin? or viomycin? or virginiamycin? or beta-lactams) adj2 (use* or misuse or consume or consumption or intake or dose or dosage or prescription* or prescrib* or overprescrib* or sale* or rate*)).ab,ti.
	5. 3 and 4
Change/impact	6. (adjust* or alter* or change or changes or changing or control* or decreas* or limit* or modify or modified or modifying or reduce or reducing or reduction* or restrict* or appropriate or inappropriate or rational* or irrational*).ab,ti.
Policy	7. exp. policy/
	8. exp. policy making/
	9. exp. legislation as Topic/
	10. exp. Government/
	11. Government Regulation/
	12. (program* or campaign* or policy or policies or guideline* or ban or banned or Regulat* or law or laws or prohibit* or restrict* or legislat* or report* or tax* or audit* or formular* or expenditure* or spending or label* or market* or advertis* or consultation*).ab,ti.
	13. (health$ adj2 (policy or policies or planning or priorit$)).ab,ti.
	14. 7 or 8 or 9 or 10 or 11 or 12 or 13
	15. 5 and 6 and 14
Humans only	16. exp. animals/ not humans.sh.
	17. 15 not 16

### Screening and eligibility

We anticipate that some studies may not clearly state in the title or abstract that the intervention is based on a government policy intervention. To ensure that these articles are not inadvertently excluded, we will conduct screening and eligibility assessment in two stages. First, at the title/abstract screening stage, articles will be assessed to determine if they meet a set of basic criteria. Second, at the full-text review stage, articles will be assessed thoroughly to determine whether the study meets the remaining inclusion/exclusion criteria. The inclusion/exclusion criteria for both stages are listed in Table 1. Conference abstracts and proceedings will be eligible for inclusion in the descriptive review if they provide sufficient detail to describe the intervention. However, given that conference abstracts often differ in major ways from the final published papers [27], they will be excluded from the analysis in the effectiveness review.

### Data management and extraction

Search results from all electronic databases will be aggregated using EndNote X7 software. De-duplication will be carried out using the methods described by Bramer et al. [28] which aims to reduce the number of false duplicates excluded while minimizing the need for manual de-duplication. Title/abstract screening, full-text review, data extraction, and risk of bias assessment will be carried out independently by two reviewers. Any disagreements between reviewers will be resolved by consensus or in consultation with a third reviewer (SJH) if needed. A PRISMA diagram will be generated to summarize the flow of studies through the stages of the review [29]. Covidence software [30] will be used for screening. Data extraction will be conducted using a customized data extraction tool that will be pilot-tested in advance. Table 3 summarizes the data that will be extracted from each included study.

**Table 3:** Data extraction fields

General information	Authors
	Publication year
	Journal
	Country of study
Study characteristics	Intervention aim
	Study aim
	Study design
	Study duration, including duration of pre-intervention period and duration of follow-up
Study participants	Description of population of interest
	Method of recruitment
	Inclusion/exclusion criteria for study participants
	Sample size calculation/power calculation
Intervention characteristics	Enacting government authority
	Description of policy intervention
	Description of intervention groups
	Number of intervention groups
	Intervention duration and timing
Data analysis	Type of analyses conducted
	Statistical tests conducted
	Confounders
Intervention effects	Baseline and post-intervention results
	Estimates of effect, including by time or subgroup as appropriate
	Secondary outcomes: beneficial outcomes
	Secondary outcomes: adverse events

### Quality assessment and risk of bias

Studies that meet the inclusion criteria for the effectiveness review will be assessed for bias using the risk of bias criteria developed by Cochrane’s EPOC group [31] which is based upon Cochrane’s Risk of Bias Tool [32] and provides advice on assessing randomized trials, controlled before-after studies, and interrupted time-series studies. Studies will be assessed with regard to selection bias, performance bias, detection bias, attrition bias, reporting bias, and other sources of bias. For controlled studies, we will examine baseline imbalances in antibiotic prescribing levels and population age and sex/gender distributions. Studies will be assessed as “low risk of bias,” “high risk of bias,” or “unclear risk of bias” on each criterion, and a summary table will be produced for each study.

### Strategy for analysis

#### Descriptive review

Data collected for the descriptive review of government policy interventions will be tabulated and summarized narratively. Articles will be categorized into seven groups by policy type based on the Behavior Change Wheel framework [23]: communication/marketing, guidelines, fiscal, regulation, legislation, environmental/social planning, and service provision. We will produce summary figures to report on the characteristics of each intervention type.

#### Effectiveness review

The government policy interventions that we identify are likely to be implemented across many populations, settings, and healthcare systems and with different goals. Some studies may aim to reduce all antimicrobial use, while others may target inappropriate antimicrobial use, or aim to reduce the use of specific antimicrobial agents. Given this diversity, it would be useful to generate estimates of effect by policy type. Therefore, if appropriate, we will conduct meta-analyses as part of our effectiveness review using the data from those studies that employed a rigorous study design. To be included in the meta-analysis, quasi-experimental studies will need to meet the minimum methodological requirements of the EPOC group. The two preferred analysis methods for interrupted time-series studies are segmented regression analysis with time trends before and after the intervention adjusting for autocorrelation and any periodic changes, or ARIMA analysis. Results for outcomes can be presented as changes along two dimensions: change in level, which is the immediate effect of the intervention, and change in slope, which is the change in the trend from pre- to post-intervention. Change in level is measured as the difference between the fitted value for the first post-intervention data point (e.g., 1 month after the intervention) minus the predicted outcome for the first post-intervention data point based on the pre-intervention slope only. Change in slope is the change in the trend from pre- to post-intervention and can be used to determine the long-term effect of the intervention. Since the interpretation of changes in slope can be difficult, we anticipate presenting the long-term effects of the interventions as proposed for the immediate effects: for example, the difference between the fitted value 6 months post-intervention (half a year after the intervention) minus the predicted outcome 6 months post-intervention based on the pre-intervention slope only. As appropriate, effects after 1 year, 2 years, etc., will be measured similarly. Where possible, we will re-analyze the results of any interrupted time-series studies that have not used these analytic strategies; we will also re-analyze the results of uncontrolled before and after studies using segmented regression analysis if the studies have at least three measurements pre- and post-intervention. Where necessary, time series data will be extracted from tables and figures using a software package that reads values from images.

If feasible and appropriate, meta-analysis will be conducted for each category of policy described in the descriptive review. We do not anticipate that studies will share a common unit of measurement; as such, individual study effect estimates will be generated for continuous outcomes using standardized mean differences, and odds ratios will be converted to standardized mean difference. A random-effects model will be used for meta-analysis. We will present forest plots to explore variation due to the inclusion of non-randomized studies. If more than 10 studies are included in the meta-analysis, we will investigate publication bias using funnel plots. Asymmetry will be assessed visually and using the Egger test [32], and additional exploratory analyses will be conducted if we identify evidence of reporting bias. If individual studies report the necessary data, we will undertake a sex- and gender-based analysis to determine whether government interventions have differential effects across these subgroups. Additionally, if we determine that there are sufficient sex- and gender-based data from studies on a specific policy (e.g., restriction of over-the-counter antimicrobial sales), we will determine whether meta-analysis is feasible in this case. Sensitivity analyses will be conducted to consider the impact of dropping weaker study designs and studies at high risk of bias.

We expect to see substantial variability across studies and will explore this using the *I* ^2^ statistic [33]. In line with the Cochrane Handbook’s guideline that an *I* ^2^ statistic > 50% may indicate substantial heterogeneity [32], we will pool studies and conduct exploratory subgroup analyses if we find an *I* ^2^ greater than this value. Planned subgroup analyses include interventions that have been implemented in low- and middle-income countries and interventions that targeted particular audiences, for example, health care providers.

Should meta-analysis not be feasible, a narrative review and structured synthesis of the evidence will be conducted using the studies with rigorous study designs. Within and across the categories of policy intervention described above, we will report on ranges of effects, heterogeneity, and quality of the evidence. If appropriate, we will also report on the intervention effects by target population (e.g., health professionals vs. general public) and, if data are available, by sex and gender.

### Missing data

Where necessary statistical data are missing (e.g., standard deviations), we will contact the study authors directly by using the email address of the corresponding author provided in the article or by searching the Internet for another email address for the corresponding author if the published address is invalid. Authors will be contacted twice before data are marked as missing. Any missing data that we are not able to obtain will be documented in the data extraction form.

If we identify cluster randomized trials that have not appropriately accounted for the unit of randomization, we will contact the authors for an estimate of the intra-cluster correlation coefficient or use external databases to impute an appropriate estimate to adjust for clustering.

### Interpretation of review findings

The Grading of Recommendations Assessment, Development and Evaluation (GRADE) system for rating the quality of evidence will be used in presenting the findings of this systematic review, as recommended by the Cochrane Collaboration [34]. The major outcomes for the effectiveness review will be presented in a summary of findings table.

## Discussion

To our knowledge, this will be the first systematic review to investigate the range of government interventions on antibiotic use and to consider the impact of government policy or regulatory action on antibiotic use. In identifying and describing government-level interventions (aim 1) using a systematic search strategy, we will be creating a valuable tool for policymakers. Since we will be conducting this search systematically, we will be maximizing our likelihood of identifying the full set of government policy interventions that have been implemented and evaluated to date. In transparently synthesizing evidence that assesses the effectiveness of these interventions, we can offer higher-quality evidence from around the world to inform the consideration, prioritization, and adoption of new government policies to reduce human antimicrobial use. The identification of any gaps in the evidence about the effectiveness of different government policy interventions will also help inform future priorities for researchers and research funders.

### Strengths and limitations

As the first systematic review to focus on government policy interventions addressing AMR, the review will provide valuable information for policymakers, public health practitioners, and researchers. Our search strategy has been carefully designed in consultation with two research librarians at the University of Ottawa—a health research librarian and a government research librarian—to incorporate databases from multiple academic disciplines and to employ several gray literature searching techniques to assist in locating relevant non-academic information sources. The protocol has been designed to meet the Cochrane Collaboration’s standards for conducting a systematic review.

We recognize the potential for publication bias in this review, particularly since much of the data we seek may not reside in easily searchable databases of peer-reviewed academic literature. We have gone to great lengths in designing this study to minimize the impact of such publication bias. First, we consulted with an additional librarian focused on government research to identify strategies for identifying government documents online. Second, we will be consulting with subject-matter experts to identify other sources. These two mechanisms, along with hand-searching reference lists of articles, will assist us in identifying the relevant literature.

A second concern for this study is data quality. Since we are investigating government policy interventions, we do not expect to identify, for example, many randomized controlled studies. Although there is a greater risk of bias associated with quasi-experimental analyses, we have chosen to include these studies in addition to randomized controlled trials in both the descriptive and effectiveness reviews. In so doing, we will identify a broader range of government policy interventions, rather than identifying only those that suit the randomized controlled design. For the effectiveness review, we have chosen to include quasi-experimental studies, but to analyze only those studies with more rigorous study designs. This way we can place greater confidence in the estimates of effectiveness in these studies as they must, at the very least, include control groups or pre-intervention data. Additionally, all studies will be analyzed for risk of bias and in accordance with GRADE quality of evidence criteria.

## Additional file

Additional file 1: PRISMA-P (Preferred Reporting Items for Systematic review and Meta-Analysis Protocols) checklist: recommended items to address in a systematic review protocol*. (DOCX 31 kb)
Additional file 2: Medline search strategy. (DOCX 161 kb)

## Abbreviations

AMR: Antimicrobial resistance
EPOC: Effective Practice and Organization of Care
GRADE: Grading of Recommendations Assessment, Development and Evaluation
PRISMA: Preferred Reporting Items for Systematic Reviews and Meta-Analyses
PROSPERO: International Prospective Register of Systematic Reviews
